# Nrf2/ARE Pathway Involved in Oxidative Stress Induced by Paraquat in Human Neural Progenitor Cells

**DOI:** 10.1155/2016/8923860

**Published:** 2015-11-15

**Authors:** Tingting Dou, Mengling Yan, Xinjin Wang, Wen Lu, Lina Zhao, Dan Lou, Chunhua Wu, Xiuli Chang, Zhijun Zhou

**Affiliations:** School of Public Health and Key Laboratory of Public Health Safety of the Ministry of Education, Fudan University, Shanghai 200032, China

## Abstract

Compelling evidences have shown that diverse environmental insults arising during early life can either directly lead to a reduction in the number of dopaminergic neurons or cause an increased susceptibility to neurons degeneration with subsequent environmental insults or with aging alone. Oxidative stress is considered the main effect of neurotoxins exposure. In this study, we investigated the oxidative stress effect of Paraquat (PQ) on immortalized human embryonic neural progenitor cells by treating them with various concentrations of PQ. We show that PQ can decrease the activity of SOD and CAT but increase MDA and LDH level. Furthermore, the activities of Cyc and caspase-9 were found increased significantly at 10 *μ*M of PQ treatment. The cytoplasmic Nrf2 protein expressions were upregulated at 10 *μ*M but fell back at 100 *μ*M. The nuclear Nrf2 protein expressions were upregulated as well as the downstream mRNA expressions of HO-1 and NQO1 in a dose-dependent manner. In addition, the proteins expression of PKC and CKII was also increased significantly even at 1 *μ*M. The results suggested that Nrf2/ARE pathway is involved in mild to moderate PQ-induced oxidative stress which is evident from dampened Nrf2 activity and low expression of antioxidant genes in PQ induced oxidative damage.

## 1. Introduction

Agricultural chemicals are becoming increasingly potent environmental threats. Paraquat (PQ; 1,1′-dimethyl-4,4′-bipyridium) is a widely used fast-acting and nonselective contact herbicide, which is mainly accumulated in the lung, resulting in widespread reports for its pulmonary toxicity [1]. PQ has also been shown to cross the blood-brain barrier and enter the brain through a neutral amino acid carrier due to its structural homology to amino acids [2, 3]. Animal experiments demonstrate that prolonged PQ exposure can result in its accumulation in different brain regions [4]. Extensive evidence demonstrates that PQ is linked to nigrostriatal damage and the emergence of Parkinson symptoms in epidemiological investigations and animal studies [5]. Importantly, compelling evidence from animal models has shown that diverse environmental factors arising during early life may either directly lead to a reduction in the number of dopamine neurons in substantia nigra or cause an increased susceptibility to degeneration of these neurons with subsequent environmental insults or with aging alone [6, 7]. Moreover, some direct evidence shows that PQ has the ability to cross the placenta and it was found in higher concentrations in the placenta than in the mother's blood [8]. Exposure to PQ in the early life can produce progressive, permanent, and cumulative neurotoxicity of the nigrostriatal dopamine system and enhance vulnerability to subsequent environmental insults [6, 7]. Our previous study in vitro suggested that PQ could reduce viability of human embryonic neural progenitor cells (hNPCs) by inducing oxidative stress and apoptosis [9]. Taken together, the persistence developmental neurotoxicity of PQ which may contribute to later-in-life adverse effects needs more attention.

Although the mechanism of PQ neurotoxicity has not been completely clear, the importance of oxidative stress in it has been generally proved [10–12]. PQ can induce oxidative damage to various neurocytes like neurons and hippocampus cells [13–15]. PQ induces oxidative stress through producing reactive oxygen species (ROS), including hydrogen peroxide, superoxide anion, and hydroxyl radicals, and consuming nicotinamide adenine dinucleotide phosphate (NADPH), an important intracellular reducing agent to exert its toxicity. In addition, ROS is an important second messenger involved in activation or regulation of cell apoptosis. In the apoptosis pathway, caspase-dependent mitochondria pathway plays a decisive role. Divergent cellular stresses promote the release of caspase-activating factors, notably cytochrome c (Cyc) from mitochondrial intermembrane space, to cytoplasm to combine with apoptotic protease activating factor-1 (Apaf-1) and adenosine triphosphate (ATP) to form apoptosome assembly, activating caspase-9 to further activate caspase-3, driving caspase cascade in cytoplasm, causing substrate proteolysis and cellular collapse to induce cell apoptosis [16, 17].

Therefore, the identification of the potential antioxidant pathway against oxidative damage had attracted intense interest. Among the multiple mechanisms, Nrf2-Keap1/ARE signal pathway is the most important endogenous antioxidant pathway discovered to date. While being attacked by ROS or other exogenous toxicant, the cytoplasm nuclear factor erythroid 2-related factor 2 (Nrf2) which was activated by dissociation with kelch-like ECH-associated protein (Keap1) entered the nucleus, binding to antioxidant reactive element (ARE), starting the transcription of corresponding downstream antioxidant molecules including phase II detoxifying enzymes [18] and antioxidative proteins [19, 20], to suppress the oxidative stress and maintain the redox balance. Several antioxidative enzymes and detoxifying enzymes levels had been downregulated in Nrf2 knockout mouse, which made the mouse more susceptive to toxin damage [21].

In addition, the developing brain is much more susceptible to being injured than the adult's brain [22, 23]. Neural stem cells' self-renewal and multipotent differentiation capacity makes it an ideal model in studying neurodevelopmental toxicological mechanism [24, 25]. In this study, we investigate the effects caused by PQ on the imbalance of oxidation and antioxidation and the role of the antioxidation pathway—the Nrf2/ARE pathway in PQ induced neurotoxicity in hNPCs.

## 2. Material and Methods

### 2.1. Chemicals and Solution

PQ was purchased from Sigma Chemical Co. (Sigma-Aldrich, Milan, Italy). ReNcell NSC Maintenance Medium and accutase were obtained commercially from Millipore (Temecula, CA). Epidermal growth factor (EGF) and basic fibroblast growth factor (FGF-2) were purchased from PeproTech. Laminin was purchased from Invitrogen (Carlsbad, CA, USA). Catalase Assay Kit, Malondialdehyde Assay Kit, Lactate Dehydrogenase Assay Kit, BCA Protein Assay Kit, Cell Lysis Buffer for Western and IP, goat anti-rabbit IgG-HRP, and goat anti-mouse IgG-HRP were obtained from Beyotime (Jiangsu, China). Total Superoxide Dismutase Assay Kit was purchased from Dojindo Molecular Technologies (Kumamoto, Japan). Tripure was obtained from Roche (Basel, Switzerland). The AMV first strand cDNA Synthesis Kit was purchased from MBI (Fermentas, Canada). Real-time PCR Kit was obtained from Tiangen Biotech (Beijing, China). Rabbit anti-Nrf2 polyclonal antibody, rabbit anti-Keap1 polyclonal antibody, rabbit anti-PKC polyclonal antibody, and rabbit anti-CKII polyclonal antibody were purchased from GeneTex (San Antonio, USA). Mouse anti-*β*-tubulin polyclonal antibody was purchased from Boster (Wuhan, China).

### 2.2. Cell Culture and PQ Treatment

Human neural progenitor cells (hNPCs) were obtained from Millipore (Temecula, CA). Frozen cells were thawed and expanded on laminin-coated 100 mm diameter dish (Corning, Inc., Corning, NY) in complete medium containing fresh EGF (20 ng/mL) and FGF-2 (20 ng/mL). Cells were passaged when they were 80% confluent. After accutase dissociation and centrifugation at 300 g for 3 min, cells were resuspended in complete medium and plated in laminin-coated dish (Corning, Inc., Corning, NY). The cultures were incubated at 37°C in 5% CO_2_. The medium was replaced every 24 h.

The cells were plated at a density of  1 × 10^5^/mL in laminin-coated plates. When the cells are approximately 70%–80% confluent, PQ dissolved in PBS was added at concentrations ranging from 0, 1, and 10 to 100 *μ*M and the cultures were maintained for 24 h.

### 2.3. Measurement of Biomarkers of Oxidative Stress

After treatment of PQ for 24 h, the levels of methane dicarboxylic aldehyde (MDA) were determined as an indicator of lipid peroxidation. And the activities of superoxide dismutase (SOD), catalase (CAT), and MDA were measured using qualified kits [26, 27]. The absorbance was obtained using a Microplate Reader (Biotek, USA) reading at corresponding wavelength.

### 2.4. Lactate Dehydrogenase (LDH) Leakage Assay

Oxidative stress-induced cytotoxicity was determined in a colorimetric assay based on the measurement of LDH released into the supernatant, which was determined using an LDH cytotoxicity assay kit according to the manufacturer's instructions [28]. The absorbance was obtained using a Microplate Reader (Biotek, USA) reading at 450 nm.

### 2.5. RNA Purification, Reverse Transcription, and Quantitative Real-Time PCR

Cell samples were collected after being exposed to PQ for 24 h. Total RNA was first extracted by Tripure following manufacturer instructions. The synthesis of the cDNA was performed utilizing an oligo(DT) primer and reverse transcriptase. All the primers were designed and synthesized based on Primer Premier Software 5.0 (PREMIER Biosoft International) by Sangon Biotech (Shanghai) Co., Ltd. The *β*-actin primers were 5′-CTCCATCCTGGCCTCGCTGT-3′ (sense) and 5′-GCTGTCACCTTCACCGTTCC-3′ (antisense; NM_001101). The HO-1 primers were 5′-TCGCCCCTGTCTACTTCC-3′ (sense) and 5′-GCAGCTCCTGCAACTCCT-3′ (antisense; NM_002133). The NQO-1 primers were 5′-GCCTAGCACAAGTACCACTCTTGGTC-3′ (sense) and 5′-CTGAGGCAGGAGAATTGCTGGAACC-3′ (antisense; NM_001025434). *β*-actin was used as the housekeeping gene. The real-time PCR was done using 2 *μ*L of cDNA, 0.5 *μ*L of each primer, and 2 *μ*L SYBR Green PCR Master Mix in a 10 *μ*L reaction volume. PCR program was a 10 min activation step at 95°C, followed by 40 cycles of 94°C for 15 s, 55°C for 30 s, and finally 72°C for 1 min. Every sample was done in triplicate. The ΔΔCt method was applied for RNA relative expression quantification [9].

### 2.6. Western Blot Analysis of Cytoplasmic Nrf2, Nuclear Nrf2, Protein Kinase C (PKC), Casein Kinase II (CKII), Cyc, and Caspase-9

After treatment with 0, 1, 10, and 100 *μ*mol/L PQ for 24 h, the protein extraction and western blot process were operated according to the previous study [9]. The immunoreactive proteins were detected by enhanced chemiluminescence using hyperfilm and enhanced chemiluminescence reagent (GE Healthcare, Little Chalfont, Buckinghamshire, UK) according to the manufacturer's instructions. Band intensities were quantified by densitometer analysis system and expressed as IOD (integrated optical density). Target protein densitometry values were adjusted to *β*-tubulin intensity and normalized to expression from the control sample.

### 2.7. Statistical Analysis

Data were analyzed with the SPSS 17.0 statistic program and expressed as means ± standard deviations (SD). All data were analyzed by one-way analysis of variance (one-way ANOVA), followed by LSD-*t* test for variance homogeneity and Kruskal-Wallis *H* test for variance heterogeneity. Data obtained at each chemical concentration were compared with the controls. *p*-value < 0.05 was considered significant.

## 3. Results

### 3.1. The Oxidative Damages Induced by PQ in hNPCs

**Figure 1:**
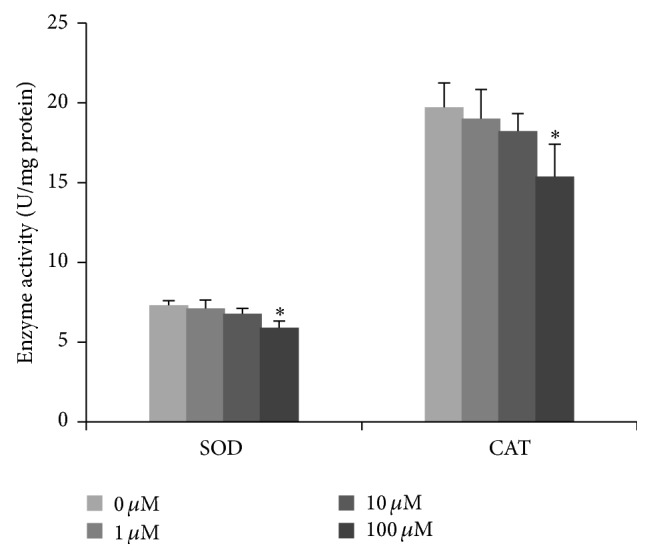
SOD and CAT activity in hNPCs upon exposure to different concentrations of PQ for 24 hr. Results are expressed as means ± S.D. (*n* = 3). *∗* means *p* < 0.05 when compared with the corresponding control group (0 *μ*M).

Previous reports from our laboratory indicated that PQ can significantly reduce cells viability to 69% at concentration of 100 *μ*M while it had no effect at 0, 1, and 10 *μ*M [9]; we chose 0, 1 and 10 *μ*M PQ which were no significant cytotoxic and 100 *μ*M PQ which was significant cytotoxic as the exposure concentration in this study. Considering the ROS generation induced by PQ at 10 *μ*M [9] and the balance between oxidation and antioxidation in normal circumstance, we first investigated the antioxidative molecules' SOD and CAT activity to research the oxidative mechanism after treatment with various concentrations of PQ. The two antioxidant enzyme activities both had a dose-dependent decrease with the significant decrease at 100 *μ*M PQ exposure compared to untreated group (*p* < 0.05) (Figure 1).

**Figure 2:**
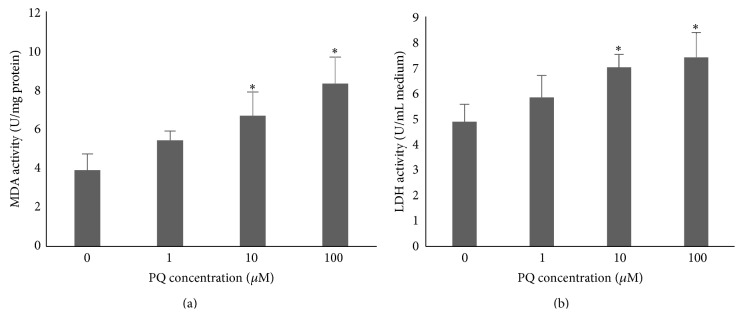
The oxidative damage of hNPCs induced by PQ treatment. (a) The intracellular MDA activity in hNPCs upon exposure to different concentrations of PQ. (b) The released LDH level of hNPCs upon exposure to different concentrations of PQ. Results are expressed as means ± S.D. (*n* = 3). *∗* means *p* < 0.05 when compared with the corresponding control group (0 *μ*M).

As the final metabolite of lipid peroxidation, MDA can change the construction and function of cell membrane, leading to the membrane breakage [29]. Besides, LDH which was released when the membrane damaged is considered a kind of sensitive markers of membrane breakage. Therefore, we detected the PQ induced oxidative damage via measuring the intracellular MDA and LDH activity. After treatment with various concentrations of PQ, we found a significant dose-dependent increase of MDA and LDH activity with PQ concentrations as low as 10 *μ*M compared to untreated group (*p* < 0.05) (Figure 2), which were accordant with ROS [9].

### 3.2. Nrf2/ARE Pathway Involved in Oxidative Stress Induced by PQ in hNPCs

**Figure 3:**
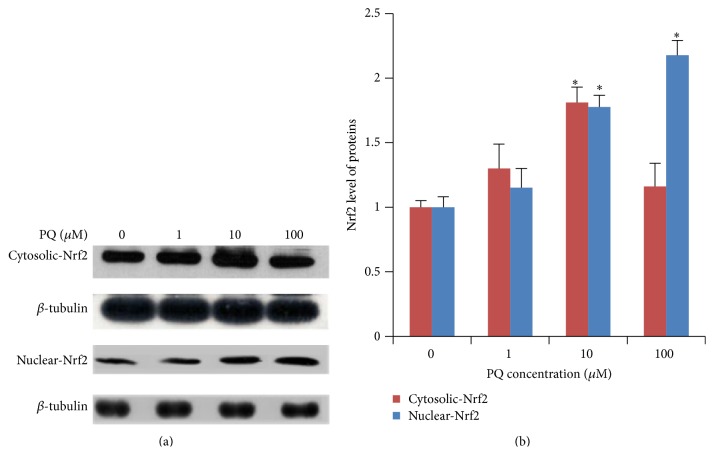
The expression of cytoplasmic and nuclear Nrf2 proteins in hNPCs after exposure to different concentrations of PQ. (a) Electrophoretic band of cytoplasmic and nuclear Nrf2 proteins by western blot. (b) Quantification of cytoplasmic and nuclear Nrf2 proteins expression. Results are expressed as means ± S.D. (*n* = 3). *∗* and *∗∗* mean *p* < 0.05 and *p* < 0.01 when compared with the corresponding control group (0 *μ*M).

Nrf2-ARE-driven genes coordinately function to protect cells from H_2_O_2_-induced apoptosis in cell studies [30].  Toxic doses of H_2_O_2_ vary with the cell density, components in culture media, and the cell type studied. The following oxidative stress may result in apoptotic and/or necrotic cell death depending on a variety of factors. To examine the potential role of Nrf2 signaling pathway in preventing PQ induced oxidative stress in hNPCs, we detected the cytoplasmic and nuclear Nrf2 protein expression by western blot. As Figure 3 showed, the cytoplasmic Nrf2 expression was significantly upregulated to 181% at 10 *μ*M of PQ (*p* < 0.05) but fell back to 116% at 100 *μ*M (*p* > 0.05). The nuclear Nrf2 expression was significantly upregulated to 178% and 218% at 10 and 100 *μ*M (*p* < 0.05).

**Figure 4:**
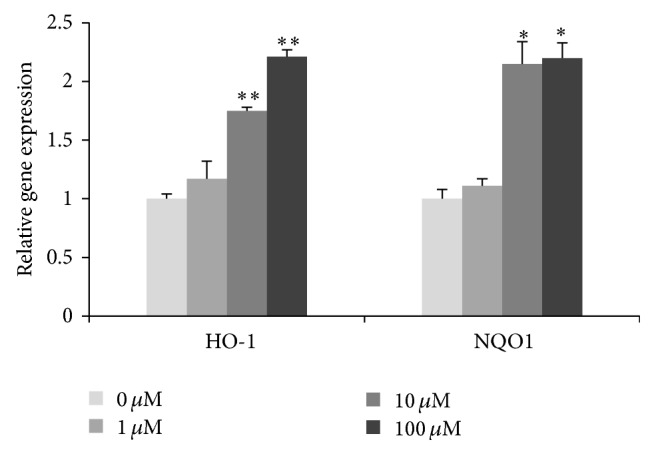
The gene expression of HO-1 and NQO1 in hNPCs after exposure to different concentrations of PQ. Results are expressed as means ± S.D. (*n* = 3). *∗* and *∗∗* mean *p* < 0.05 and *p* < 0.01 when compared with the corresponding control group (0 *μ*M).

As a result of nuclear Nrf2 increase, we examine Nrf2-ARE-driven genes using real-time PCR after PQ treatment for 24 h. HO-1 mRNA expression was slightly increased to 117% at 1 *μ*M and significantly increased to 175% and 221% at 10 and 100 *μ*M (*p* < 0.05), respectively. NQO1 expression was slightly increased to 111% at 1 *μ*M and significantly increased to 215% and 220% at 10 and 100 *μ*M (*p* < 0.01) (Figure 4).

### 3.3. Activation of Protein Kinase (PKC and CKII) Induced by PQ in hNPCs

**Figure 5:**
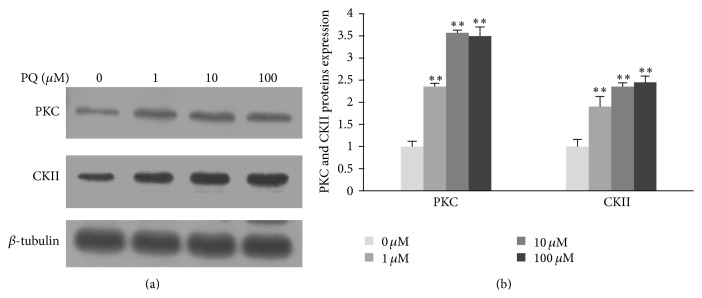
The expression of PKC and CKII proteins in hNPCs after exposure to different concentrations of PQ. (a) Electrophoretic band of PKC and CKII proteins by western blot. (b) Quantification of PKC and CKII proteins expression. Results are expressed as means ± S.D. (*n* = 3). *∗∗* means *p* < 0.01 when compared with the corresponding control group (0 *μ*M).

Previous studies had discovered the involvement of protein kinase in phosphorylating Nrf2 and triggering its nuclear translocation in response to oxidative stress [31]. In our study, we used western blot to detect the intracellular PKC and CKII proteins expression after treatment with different concentrations of PQ for 24 h to observe the role of protein kinases in PQ induced Nrf2 activation. As Figure 5 showed, the intracellular PKC and CKII expressions were both significantly upregulated at even 1 *μ*M of PQ (*p* < 0.01).

### 3.4. Effect of PQ on Apoptotic Cell Death Signaling Pathways of hNPCs

**Figure 6:**
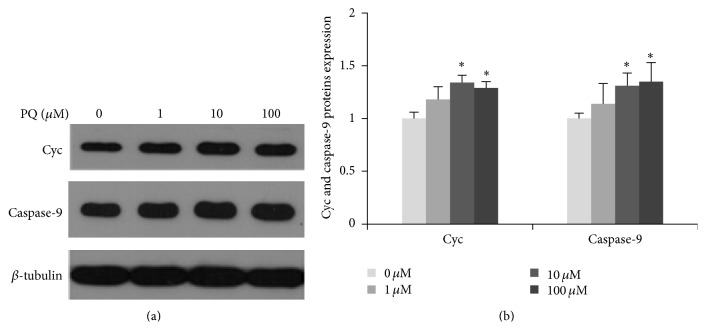
The expression of Cyc and caspase-9 proteins in hNPCs after exposure to different concentrations of PQ. (a) Electrophoretic band of Cyc and caspase-9 proteins by western blot. (b) Quantification of Cyc and caspase-9 proteins expression. Results are expressed as means ± S.D (*n* = 3). *∗* means *p* < 0.05 when compared with the corresponding control group (0 *μ*M).

Oxidative stress had been shown to activate caspase-dependent apoptotic cell death signaling pathways [32]. Cyc combined with Apaf-1 and subsequently activated caspase-9 to initiate downstream effector caspases to cause the cell apoptosis [33, 34]. Herein, we examined the protein expressions of Cyc and caspase-9.  Figure 6 showed that Cyc and caspase-9 protein expressions were both significantly increased in 10 *μ*M PQ treatment group compared with the control (*p* < 0.05).

## 4. Discussion

In the present study, we demonstrate that PQ can directly produce toxicity to hNPCs by inducing ROS generation and decreasing SOD and CAT activity which resulted in redox imbalance and oxidative damage. In particular, we found that PQ induced oxidative stress can activate the Nrf2-Keap1/ARE signaling pathway to initiate the downstream antioxidant responsive elements including HO-1 and NQO1 mRNAs expression to prevent the oxidative damage. Additionally, we observed that PQ can activate PKC and CKII which were involved in the phosphorylation of Nrf2, revealing that PKC and CKII may play an indirect part in antioxidative stress.

As one of the most widely used herbicides in the world, PQ can induce damage to various organs or cells [35–37]. To the nervous system, because of the structural similarity to the parkinsonism-inducing neurotoxic agent 1-methyl-4-phenyl-1, 2, 3, 6-tetrahydropyridine (MPTP), PQ is considered to be a possible environmental risk factor for neurodegenerative disorders like Parkinson's disease (PD) [38, 39]. In addition, the developing brain is much more susceptible to be injured than the adult's brain [21, 22]. Therefore, PQ developmental neurotoxicity deserves more attention. It was reported that PQ could inhibit the proliferation and disrupt the differentiation of neural precursor cells in vitro studies [40]. Also, in our previous study, we found the concentration of PQ reducing the hNPCs viability (100 *μ*M) [9] was lower than other cell types [41, 42], suggesting the sensitivity of hNPCs to PQ induced toxicity. Based on the effect of PQ on hNPCs viability, in this study, we chose 0, 1, 10, and 100 *μ*M as the exposure concentrations.

Because of its extensive effects on cell proliferation, differentiation, apoptosis, and signal transduction, ROS plays an important role in neurotoxicity. Several studies have suggested that PQ could cause dopaminergic neurons and hippocampal neurons damage via generating ROS to cause oxidative damage to brain mitochondria [15, 43]. Our previous study had also shown that PQ exposure could cause a dose-dependent increase in ROS production and significant increases were observed when PQ doses were increased to 10 *μ*M [9]. Besides, LDH which is released when the membrane damaged is considered a kind of sensitive markers of membrane breakage. Similarly, our study showed that PQ increased the LDH and MDA levels. These results suggest that PQ induced the production of lipid peroxidation and oxidative damage in hNCPs.

As a sensitive receptor for oxidative stress, Nrf2/Keap1 signaling pathway played a crucial role in preventing cells from apoptosis, stress, inflammation, and tumor [20]. It was the most important intrinsic antioxidative stress pathway yet discovered [44, 45]. When the Nrf2/Keap1 signaling pathway was activated, Nrf2 was uncoupled from inhibitor protein-Keap1 and accessed to nucleus to bind with ARE, leading to the transcription of the downstream target antioxidative genes, and sequentially improved cell's antioxidative stress ability [46, 47]. Similarly, in our study, 10 *μ*M PQ induced oxidative stress can significantly activate the Nrf2 pathway to prevent the oxidative stress via increasing the level of both cytoplasmic and nuclear Nrf2 protein and the Nrf2 downstream antioxidative genes, HO-1 and NQO1, which meant the activation of Nrf2/Keap1 signaling pathway to increase nuclear antioxidative genes expression to prevent 10 *μ*M PQ caused oxidative stress.

In other way, normal cells can eliminate the redundant ROS through antioxidant enzyme including SOD and CAT to prevent the oxidative damage. But severe exposure to toxicant could increase the oxidation but decrease the antioxidation to damage the oxidative balance. Previous studies have showed that SOD and CAT in dopaminergic neurons were all decreased after PQ exposure [41, 48]. Similarly, our study showed that higher level of PQ (100 *μ*M) can significantly inhibit antioxidant enzymes SOD and CAT activity, which meant the imbalance of oxidation and the severity of 100 *μ*M PQ induced oxidative damage. Enhanced nuclear Nrf2 expression was further augmented by PQ treatment to further activate downstream antioxidative genes HO-1 and NQO1 expressions to defend the oxidative damage. The level of cytoplasmic Nrf2 fell back at 100 *μ*M. Similarly, a previous study also has discovered that higher concentration of PQ exposure (0.5 mmol/L) could inhibit neuroblastoma Nrf2 protein level [49].

In addition, some protein kinases like PKC and CKII could also induce the Nrf2 protein phosphorylation to influence its activity [31, 50]. In our study, the intracellular PKC and CKII protein levels were elevated with the increasing of PQ concentration even at 1 *μ*M, which revealed the correlation between Nrf2 activation and its phosphorylation caused by PKC and CKII.

However, as an important second messenger involved in regulation of cell apoptosis, high level of ROS induced by PQ could inevitably cause mitochondria damage and cell apoptosis [51, 52]. In our study, the mitochondria released Cyc level increased at 10 *μ*M, which resulted in the increase of caspase-9. That was consistent with the cells apoptosis and cells viability alteration discovered in previous study [9], suggesting the obvious cells damage caused by high concentration (100 *μ*M) of PQ.

In general, PQ exposure could significantly induce oxidative stress and cause oxidative imbalance. Low concentration (10 *μ*M) of PQ caused moderate stress could activate Nrf2 pathway to prevent cells from PQ induced oxidative damage; however, the Nrf2 pathway protection was ineffective in high concentration (100 *μ*M) and caused severe stress, which has finally resulted in decrease of cell ability and increase of cell apoptosis [9]. Considering the protection of Nrf2 pathway in PQ induced damage, it may be supposed as a potential and effective therapeutic target for oxidative damage related disease in clinical therapy.

## 5. Conclusion

We provide important evidence suggesting that PQ has direct toxicity to cause irreversible apoptosis to hNPCs which was associated with the elevated oxidative stress. In addition, PQ induced oxidative stress and redox imbalance could activate the Nrf2/ARE signaling pathway to prevent the oxidative stress via initiating the downstream antioxidant responsive element like HO-1 and NQO1 mRNAs expression. In particular, we also observed that PKC and CKII may be involved in the phosphorylation of Nrf2, revealing that PKC and CKII may play an indirect role in antioxidative stress.
